# Platelet-derived Growth Factor Primes Cancer-associated Fibroblasts for Apoptosis*

**DOI:** 10.1074/jbc.M114.563064

**Published:** 2020-09-09

**Authors:** Sumera I. Ilyas, Joachim C. Mertens, Steven F. Bronk, Petra Hirsova, Haiming Dai, Lewis R. Roberts, Scott H. Kaufmann, Gregory J. Gores

**Affiliations:** ‡ Division of Gastroenterology and Hepatology, Mayo Clinic, Rochester, Minnesota 55905,; § Division of Gastroenterology and Hepatology, University Hospital Zurich, Zurich 8091, Switzerland, and; ¶ Division of Oncology Research, Mayo Clinic, Rochester, Minnesota 55905

## Abstract

Desmoplastic malignancies such as cholangiocarcinoma (CCA) are characterized by a dense stroma containing an abundance of myofibroblasts termed cancer-associated fibroblasts (CAF). The CAF phenotype represents an “activated state” in which cells are primed for cell death triggered by BH3 mimetics. Accordingly, this primed state may be therapeutically exploited. To elucidate the mechanisms underlying this poorly understood apoptotic priming, we examined the role of platelet-derived growth factor (PDGF) in CAF priming for cell death given its prominent role in CAF activation. PDGF isomers PDGF-B and PDGF-D are abundantly expressed in CCA cells derived from human specimens. Either isomer sensitizes myofibroblasts to cell death triggered by BH3 mimetics. Similar apoptotic sensitization was observed with co-culture of myofibroblasts and CCA cells. Profiling of Bcl-2 proteins expressed by PDGF-primed myofibroblasts demonstrated an increase in cellular levels of Puma. PDGF-mediated increases in cellular Puma levels induced proapoptotic changes in Bak, which resulted in its binding to Bcl-2. Short hairpin RNA-mediated down-regulation of Puma conferred resistance to PDGF-mediated apoptotic priming. Conversely, the BH3 mimetic navitoclax disrupted Bcl-2/Bak heterodimers, allowing Bak to execute the cell death program. Treatment with a Bcl-2-specific BH3 mimetic, ABT-199, reduced tumor formation and tumor burden in a murine model of cholangiocarcinoma. Collectively, these findings indicate that apoptotic priming of CAF by PDGF occurs via Puma-mediated Bak activation, which can be converted to active full-blown apoptosis by navitoclax or ABT-199 for therapeutic benefit.

## Introduction

Much of the effort in cancer biology and therapy has focused on directly targeting and eradicating neoplastically transformed cells. However, achieving effective cancer cell-directed therapy has been challenging because of the diverse alterations in these transformed cells and the continued accumulation of abnormalities due to genomic instability (1, 2). In contrast, the tumor stroma appears to be more genetically uniform and represents an additional therapeutic target (1, 3., 4., 5.). Exploiting the dependence of cancer cells on the tumor microenvironment will require a further in-depth understanding of the dynamic reciprocal interaction between cancer cells and the surrounding stroma.

The hallmark of desmoplastic tumors is a dense stroma characterized by an abundance of α-smooth muscle actin (α-SMA)^4^-positive myofibroblasts termed cancer-associated fibroblasts (CAF), which play a pivotal role in cancer progression (6., 7., 8.). This dense stroma, which is found in a number of solid tumors, including cholangiocarcinoma, pancreatic cancer, and certain subtypes of breast cancer, has been proposed as a potential target for anticancer therapy (3, 6, 9, 10).

Increased apoptotic sensitivity has been well described in several “activated” cell states, including CAF (3, 11, 12). For instance, activated T-cells at the end of an immune response and activated hepatic stellate cells (HSC) in fibrotic liver injury have an increased susceptibility to cell death (11, 13). Indeed, myofibroblast apoptosis as well as their reversal to a quiescent phenotype is associated with regression of liver fibrosis (11, 12). This increased apoptotic sensitivity has been linked to enhanced ability to activate the mitochondrial pathway of apoptosis, a phenomenon termed “mitochondrial priming” (14, 15).

The mitochondrial apoptotic pathway is regulated by the Bcl-2 family of proteins. Activation of Bak or Bax, two of the proapoptotic members of this family, results in their oligomerization, insertion in the outer mitochondrial membrane, and permeabilization of that membrane to allow release of cytochrome *c* and other apoptogenic proteins from the mitochondrial intermembrane space into the cytosol (16). Antiapoptotic members of this family, including Bcl-2, Bcl-x_L_, and Mcl-1, act in part by binding Bak or Bax and preventing their oligomerization. BH3-only proteins such as Puma, Bim, Bid, Bad, and Noxa, which share only the 15-amino acid BH3 homology domain with the other Bcl-2 proteins, act as initiators of cell death by directly activating Bak and Bax or by neutralizing antiapoptotic proteins (17).

When cells are “primed” for apoptosis, BH3 mimetics (small, organic compounds that function like BH3-only proteins to bind antiapoptotic Bcl-2 family members and displace BH3-only proteins from them) are readily able to induce apoptosis. For example, we recently reported that CAF from cholangiocarcinoma (CCA), a prototypic desmoplastic cancer for the study of CAF biology (6), are primed for apoptosis (3). In particular, the BH3 mimetic navitoclax is able to not only kill these myofibroblasts *ex vivo* but also inhibit tumor growth and metastasis in a syngeneic orthotopic transplantation animal model of CCA by depleting CAF from the tumor microenvironment (3). The mechanistic basis for the sensitivity of CAF to BH3 mimetics, however, is unknown.

Platelet-derived growth factor (PDGF) is one of the most abundant growth factors in the CCA tumor microenvironment secreted by tumor cells (19). PDGF family members, particularly PDGF-B and PDGF-D, have been implicated in promoting proliferation, survival, and CAF migration in these desmoplastic tumors (19., 20., 21.). These important effects of PDGF in CAF prompted us to examine whether PDGF also participates in priming. Herein, we report that PDGF induces priming of myofibroblasts but not CCA cells. We further demonstrate that PDGF increases cellular Puma levels in myofibroblasts, leading to Puma-dependent binding of Bak to Bcl-2. BH3 mimetics that disrupt this Puma-initiated Bak/Bcl-2 interaction cause Bak-mediated cell death of the CAF. These observations provide a mechanistic basis for mitochondrial “priming” of CAF in CCA and other desmoplastic tumors.

## EXPERIMENTAL PROCEDURES

### Cell Culture

The human CCA cell lines KMCH (22), KMBC (23), HuCCT-1 (24), and Mz-ChA-1 (25), the human hepatic myofibroblast LX-2 (26), quiescent human fibroblasts (hFB) obtained from ATCC, and HSCs kindly provided by V. H. Shah (Mayo Clinic, Rochester, MN), were cultured in Dulbecco's modified Eagle's medium supplemented with 10% fetal bovine serum, penicillin (100 units/ml) and streptomycin (100 μg/ml) under standard conditions. The non-malignant SV40-immortalized human cholangiocyte cell line H69 was maintained as previously described (27, 28).

### Materials

Navitoclax and ABT-199 (Active Biochem, Hong Kong) were dissolved in dimethyl sulfoxide and added to cells in a final concentration of 1 μm from 1000-fold concentrated stocks. Recombinant human PDGF-B, recombinant human PDGF-D, and recombinant human hepatocyte growth factor (R&D Systems, Minneapolis, MN) as well as recombinant human connective tissue growth factor (PeproTech, Rocky Hill, NJ) were reconstituted according to the manufacturers' protocols and added to cells at a final concentration of 50 ng/ml. Anti-human PDGFRB (R&D Systems) was added to cells at a final concentration of 20 μg/ml.

### Immunofluorescence in Human Cholangiocarcinoma Samples

Frozen tissue samples of human CCA and corresponding samples of normal liver tissue (*n* = 12) were obtained with Institutional Review Board approval. 5-μm frozen sections were prepared on a cryomicrotome (Leica, Buffalo Grove, IL), air-dried, and stored at −80 °C. Tissue sections were fixed for 10 min in 100% acetone at −20 °C, blocked for 1 h at room temperature with calcium- and magnesium-free Dulbecco's phosphate-buffered saline (PBS) containing 5% bovine serum albumin (BSA) and 0.1% glycine, and subsequently incubated with primary antibody for 12 h at 4 °C. Antibodies were diluted in PBS containing 5% BSA and 0.1% glycine. Primary antibodies used were α-SMA (1:300; Abcam, ab7817), CK7 (1:300; Abcam, ab9021), PDGF-B (1:300; Abcam, ab21234), and PDGF-D (1:300; Abnova, H00080310-D01). After washing, slides were incubated with Alexa Fluor® 488 anti-rabbit IgG (PDGF-B and PDGF-D; 1:1000; Invitrogen, A21441) or Texas Red®-X anti-mouse IgG (CK7, α-SMA; 1:1000; Invitrogen, T6390) for 1 h in the dark at room temperature, washed again, and mounted using Prolong Antifade with 4′,6-diamidino-2-phenylindole (DAPI; Invitrogen) to visualize the nuclei. The slides were analyzed on an LSM780 confocal microscope (Zeiss, Jena, Germany) equipped with an ultraviolet laser.

### mRNA Analysis

For gene expression studies, mRNA was isolated from fresh-frozen tissue sections and cells using the RNeasy Plus Mini Kit (Qiagen, Hilden, Germany). Reverse transcription was performed using the Moloney murine leukemia virus reverse transcriptase and random primers (Invitrogen). Real time polymerase chain reaction (PCR; Light Cycler, Roche Applied Science) for quantification of the cDNA template was performed using SYBR Green (Roche Applied Science) as the fluorophore (29). The primers used in PCR reactions were: PDGF-B forward (5′-CTGGCATGCAAGTGTGAGAC-3′) and PDGF-B reverse (5′-CGAATGGTCACCCGAGTT-3′); PDGF-D forward (5′-CCATGACCGGAAGTCAAAAG-3′) and PDGF-D reverse (5′-ATTCCTGGGAGTGCAACTGT-3′); Puma forward (5′-GACCTCAACGCACAGTACGA-3′) and Puma reverse (5′-GAGATTGTACAGGACCCTCCA-3′). Expression was normalized to 18 S rRNA (Ambion, Austin, TX). Relative gene expression was calculated according to the ΔΔ CT method.

### Quantification of Apoptosis

Cells were grown to subconfluency in 96-well plates, and respective treatments were subsequently added. Cellular nuclear morphology was assessed by fluorescent microscopy after staining with DAPI (Sigma), and apoptosis was quantified as previously described (30). Caspase-3/7 activity was quantified utilizing a commercially available assay (Apo-ONE homogeneous caspase-3/7 assay, Promega Corp., Madison, WI) and a multi-detection microplate reader (Biotek, Winooski, VT).

### Generation of Stable Transfectants

HEK 293T cells were transfected with pCMV-VSV-G (Addgene), pCMV-dR8.2 dvpr (Addgene), and the lentiviral shPuma (Sigma, NM_014417.2-719; Sigma, NM_014417.2-785) and shPDGFRB constructs (Sigma, NM_002609.3-3724; Sigma, NM_002609.x-2371), respectively, using Lipofectamine LTX reagent (Invitrogen) to package the shPuma and shPDGFRB containing lentiviruses. Medium was passed through a 0.45-μm pore filter, and Polybrene (Sigma) was then added to a final concentration of 8 μg/ml. Target HSC cells grown to 50% confluency were incubated with lentivirus-containing medium from the HEK 293T cells for 3 h before medium was replaced with fresh noninfectious medium. Infection was again repeated 24 h after the initial exposure. Infected HSC were split into selection medium containing 2.5 μg/ml puromycin. Cell lysates were prepared from shPuma and shPDGFRB cells to confirm knockdown of shPuma and shPDGFRB protein by Western blotting. Stably transfected shBax and shBak clones were generated as previously described in detail (3).

### Co-culture and Conditioned Medium Experiments

Cell co-culture experiments were performed using a Transwell® insert co-culture system (24 wells) equipped with 0.4-μm pore size polyester inserts (Corning Costar, Acton, MA) according to the manufacturer's recommendations. Briefly, HSC, hFB, and shPDGFRB-HSC cells were plated alone or with human CCA cell lines in the transwell insert co-culture system (HSC, hFB, or shPDGFRB-HSC in the bottom wells and KMCH, KMBC, HuCCT-1, Mz-ChA-1, or H69 in the inserts). Initially, all cells were plated alone overnight. The co-culture insert chambers containing the CCA cell lines were then transferred the next day. After 24 h, cells were treated with vehicle or navitoclax, 1 μm. After 48 h, cells in the bottom wells were analyzed for apoptosis using DAPI staining as described above. Caspase-3/7 assay was performed utilizing conditioned medium harvested from CCA cell lines. Briefly, HSC, hFB, and shPDGFRB-HSC were plated in 96-well plates. After 24 h, conditioned medium from CCA cell lines (KMCH, KMBC, HuCCT-1, Mz-ChA-1) was harvested, passed through a 0.45-μm pore filter, and added to respective wells in the plates containing HSC, hFB, or shPDGFRB-HSC. After 24 h, cells were treated with vehicle or navitoclax 1 μm. After 48 h of navitoclax treatment, apoptosis was assessed by quantification of caspase-3/7 activity as described above.

### Immunoblot Analysis

Using whole-cell lysates prepared as previously detailed (31), proteins were resolved by SDS-PAGE and transferred to nitrocellulose or polyvinylidene difluoride membranes depending on the protein of interest. Membranes were blotted with primary antibody at the indicated dilutions. Antibody sources are as follows: Bak (G-23; 1:500), Bax (N-20; 1:500), Mcl-1 (S-19; 1:500), and actin (C-11; 1:500) from Santa Cruz Biotechnology (Santa Cruz, CA); Bid (AF860; 1:500) from R&D Systems; Bim (2819S; 1:500) and Bcl-x_L_ (54H6; 1:500) from Cell Signaling (Danvers, MA); Bcl-2 (clone 124; 1:300) from DAKO (Carpinteria, CA); Puma (PRS3041; 1:500) from Sigma. Horseradish peroxidase-conjugated secondary antibodies for mouse, rabbit, and goat (1:3000) were obtained from Santa Cruz (Santa Cruz, CA), and fluorochrome-labeled secondary antibodies for mouse, rabbit, and goat (1:10000) were from LICOR (Lincoln, NE). Proteins were visualized with enhanced chemiluminescence reagents (ECL/ECL Plus, Amersham Biosciences) and Kodak X-Omat film or by Odyssey (LICOR) infrared scanning.

### Analytical Gel Filtration

Cells were grown to confluence, and respective treatments were added. Cells were lysed using CHAPS buffer (1% CHAPS, 1% glycerol, 150 mm NaCl, 5 mm DTT, and 20 mm HEPES, pH 7.5). 200-μl aliquots of cell lysates were subjected to fast protein liquid chromatography (FPLC) at 4 °C on Superdex S200 (GE Healthcare) as previously described (32). 500-μl fractions were analyzed by SDS-PAGE followed by immunoblot analysis.

### Immunoprecipitation of Bcl-2

HSC were grown on 15-cm tissue culture dishes to subconfluency, and respective treatments were added. Cells were subsequently lysed in cold CHAPS lysis buffer (1% CHAPS, 150 mm NaCl, 20 mm HEPES, 1% glycerol, 3% thiodiglycol, 1 mm EGTA, 1 mm sodium orthovanadate, 10 mm sodium pyrophosphate, 1 mm PMSF, 1× protease inhibitor mix, 100 mm sodium fluoride, 25 nm microcystin). Lysates were centrifuged for 15 min at 15,000 × *g* to pellet cellular debris. Protein concentration was determined via Bradford assay. 400 μg of total protein was precleared by incubation with 40 μl of protein G Sepharose beads (GE Healthcare) for 1 h at 4 °C. Beads cross-linked to anti-Bcl-2 (clone 124; DAKO) or control mouse IgG (Santa Cruz) were prepared as previously described (33). 40 μl of control IgG or Bcl-2 cross-linked beads were added to the precleared 400 μg of total lysate protein and incubated rotating overnight at 4 °C. After sedimentation at 8000 × *g* for 2 min, beads were rapidly washed 4 times with CHAPS lysis buffer, resuspended in sample buffer (4 m urea, 2% (w/v) SDS, 62.5 mm Tris-HCl, pH 6.8, 1 mm EDTA, and 5% (v/v) 2-mercaptoethanol), and heated for 20 min at 65 °C to release bound polypeptides. Immunoprecipitated Bcl-2 and 1/5 dilution of input were subjected to SDS-PAGE, transferred to nitrocellulose membrane, and probed with antibodies to Bak and Bcl-2 as indicated in the figure legends.

### RNA Interference

A small interfering RNA (siRNA) (Origene, Rockville, MD) was employed to silence Bcl-2. Cells transfected with scramble siRNA were used as control. Cells were grown in 24-well plates and transiently transfected with 30 nm siRNA using siTran 1.0 (Origene) in a total transfection volume of 600 μl of Opti-MEM I (Invitrogen). Respective treatments were added after 24 h.

### Murine Model of Cholangiocarcinoma

All animal experiments were performed in accordance with a protocol approved by the Mayo Clinic Institutional Animal Care and Use Committee. A murine model of CCA, driven by sleeping beauty transposase-mediated biliary introduction of constitutively active AKT (myr-AKT) and Yes-associated protein (YAPS127A) (34) followed by interleukin-33 (IL-33) administration, was used (35). Each animal was given intraperitoneal injections of 1 μg of IL-33 (R&D Systems) starting on post-operative day 1 for 3 days. From week 4 to week 6, mice received either ABT-199 (50 mg/kg/day) or vehicle via daily oral gavage. Animals were euthanized at the end of 10 weeks and examined for the presence of tumor and metastases.

### Immunohistochemistry and Terminal Deoxynucleotidyltransferase dUTP Nick-end Labeling (TUNEL) Assay in Mice Liver Specimens

Liver tissue from euthanized mice was fixed in 4% paraformaldehyde for 48 h, embedded in paraffin, and sectioned into 3.5-μm slices. Paraformaldehyde-fixed paraffin-embedded liver tissue sections were deparaffinized, hydrated, and incubated with antibody against α-SMA (Abcam; 1:500) overnight at 4 °C. Bound antibodies were detected with biotin-conjugated secondary antibodies and diaminobenzidine (Vector Laboratories, Burlingame, CA) as a substrate, and the tissue slices were counterstained with hematoxylin. The fluorescent TUNEL assay on liver tissue (*In situ* cell death detection kit; Roche Applied Science) was performed on paraffin-embedded liver tissue sections. Briefly, paraformaldehyde-fixed paraffin-embedded liver tissue sections were deparaffinized and hydrated. The TUNEL assay was then performed using the manufacturer's protocol, and tissue slices were mounted with ProLong Gold antifade reagent with DAPI (Invitrogen). Apoptotic cells were quantified by counting TUNEL-positive nuclei in 10 random microscopic fields (63×) using the LSM780 confocal microscope (Zeiss, Jena, Germany).

### Biochemical Analysis

Serum alanine aminotransferase activity was determined using a standardized and automated procedure of the diagnostic laboratory of the Mayo Clinic.

### Statistical Analysis

Data represent at least three independent experiments and are expressed as the mean ± S.E. Differences in experiments with two groups were compared using the two-tailed Student's *t* test or the Fisher's exact test. Differences were considered as significant at levels of *p* < 0.05.

## RESULTS

### PDGF Is Abundantly Expressed in Human CCA Specimens

**FIGURE 1.**
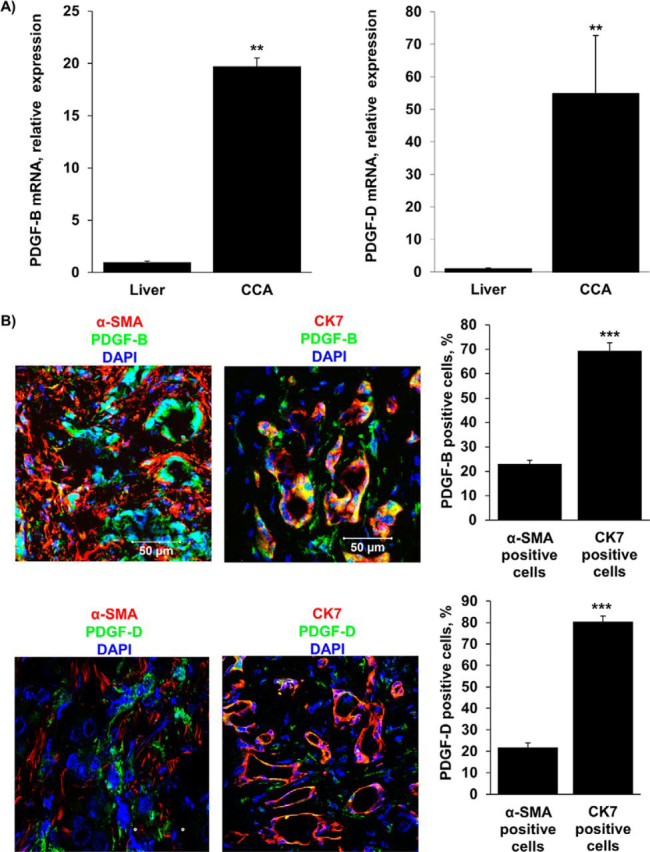
PDGF-B and PDGF-D are increased in CCA cells in human resected specimens. *A*, expression of PDGF-B and PDGF-D was quantified in human resected CCA specimens and the corresponding normal livers by quantitative real-time-PCR. Mean ± S.E. are depicted for *n* = 12; **, *p* < 0.01. *B*, immunofluorescence was used to detect PDGF-B and PDGF-D expression in CCA cells (CK7-positive) compared with CAF (α-SMA-positive) (*left panel*). The number of PDGF-B or PDGF-D positive CCA cells and CAF was quantified in five high power fields and expressed as a percentage of total (*right panel*). Means ± S.E. are depicted for *n* = 5. ***, *p* < 0.001. Original magnification, 63×.

PDGF family members, particularly PDGF-B and PDGF-D, have been implicated in promoting proliferation, survival, and CAF migration in desmoplastic tumors (19., 20., 21.). Initially, we sought to identify which PDGF isomer is prominently expressed in CCA. Quantitative RT-PCR demonstrated that both PDGF-B and PDGF-D mRNA are abundantly expressed in all human CCA samples as compared with corresponding non-tumor liver tissue (Fig. 1*A*). In further experiments, we examined the cellular distribution of these isomers within the CCA tissue by immunofluorescence (Fig. 1*B*). Co-staining with α-SMA and CK7, which were used to identify activated CAF and CCA cells, respectively, demonstrated that PDGF-B and PDGF-D are localized predominantly in the CCA cells and to a lesser extent in CAF. These observations indicate that both isoforms are potentially important in CCA biology.

### PDGF Selectively Sensitizes Myofibroblasts to Navitoclax-triggered Apoptosis

**FIGURE 2.**
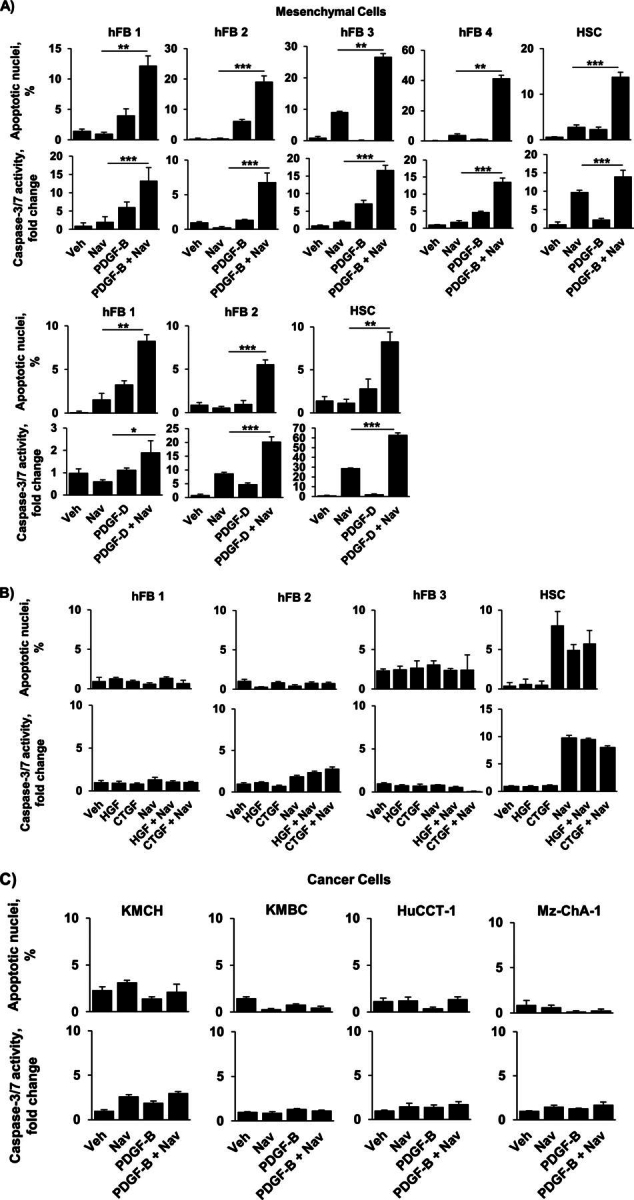
PDGF-B and PDGF-D sensitize hFB and HSC, but not CCA cells, to navitoclax-induced apoptosis. *A*, HSC and four different types of quiescent hFB (*hFB 1–4*) were treated with vehicle, navitoclax for 48 h, and PDGF-B for 24 h or PDGF-B for 24 h followed by the addition of navitoclax for 48 h. Similarly, HSC cells and two hFB cell types were treated with vehicle (*Veh*), navitoclax (*Nav*), PDGF-D, or PDGF-D for 24 h followed by the addition of navitoclax for 48 h. Apoptosis was quantified morphologically using DAPI staining plus fluorescence microscopy (*upper panels*) and biochemically by measuring caspase-3/7 activity (*lower panels*). Mean ± S.E. are depicted for *n* = 3. **. *p* < 0.01; ***, *p* < 0.001. *B*, hFB and HSC were treated with vehicle, hepatocyte growth factor (*HGF*), connective tissue growth factor (*CTGF*), and navitoclax for 24 h followed by the addition of navitoclax for 48 h or connective tissue growth factor for 24 h followed by the addition of navitoclax for 48 h. Apoptosis was quantified using DAPI plus fluorescence microscopy (*upper panels*) and by measuring caspase-3/7 activity (*lower panels*). Mean ± S.E. are depicted for *n* = 3. *C*, four different human CCA cell lines (KMCH, KMBC, HuCCT-1, Mz-ChA-1) were treated as described in *A*. Apoptosis was quantified using DAPI plus fluorescence microscopy (*upper panels*) and by measuring caspase-3/7 activity (*lower panels*). Mean ± S.E. are depicted for *n* = 3.

CAF are likely derived from several different cell types, including portal fibroblasts, HSC, and perhaps even bone marrow-derived precursor cells (36., 37., 38.). Therefore, we stimulated both hFB and HSC to obtain the activated myofibroblast state. Cells were pretreated with the growth factors PDGF-B, PDGF-D, hepatocyte growth factor, and connective tissue growth factor (all known to be expressed in CCA (6, 19, 21)) to achieve an activated phenotype and subsequently exposed to the BH3 mimetic navitoclax. Pretreatment with PDGF-B and PDGF-D, but not hepatocyte growth factor or connective tissue growth factor, significantly increased the sensitivity of hFB and HSC to navitoclax-mediated apoptosis as assessed morphologically (Fig. 2, *A* and *B*, *upper panels*) and biochemically (Fig. 2, *A* and *B*, *lower panels*). In comparison, navitoclax alone did not induce apoptosis in quiescent hFB. HSC, which represent a semiactivated state, displayed modest navitoclax sensitivity without growth factors but were further sensitized by PDGF-B or PDGF-D pretreatment. In contrast to the myofibroblast cells, human CCA cells did not exhibit apoptotic sensitization to navitoclax after PDGF treatment (Fig. 2*C*), likely due to their known overexpression of Mcl-1, which does not bind navitoclax (3). Given that either PDGF-B or PDGF-D primed myofibroblasts for cell killing by navitoclax, we elected to use PDGF-B for the preponderance of our remaining studies.

### Co-culture of Myofibroblast Cells with CCA Cells Induces Apoptotic Priming

**FIGURE 3.**
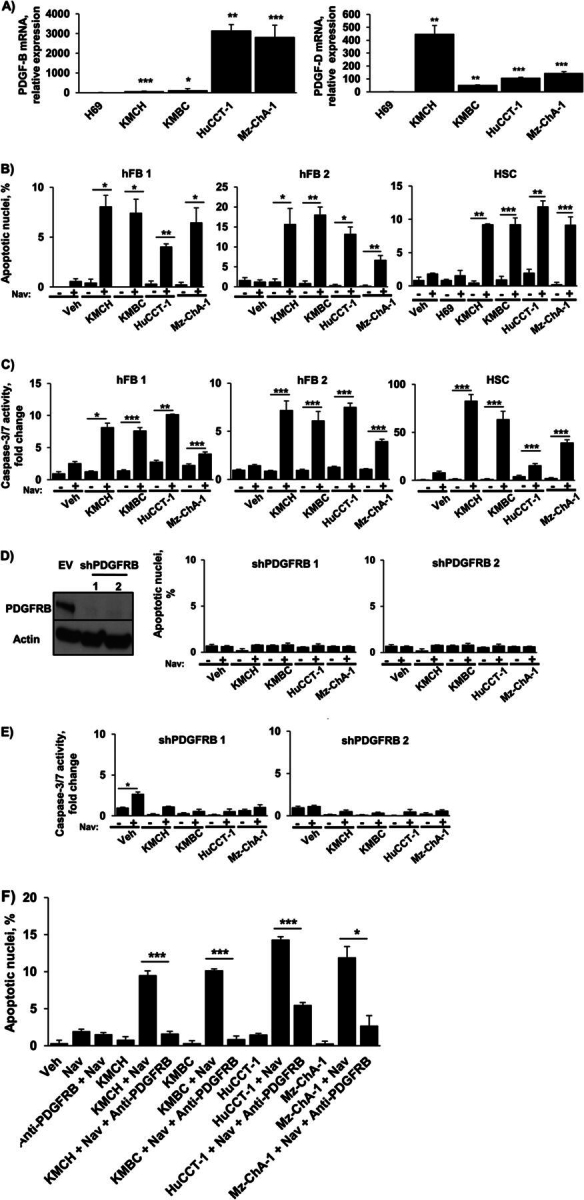
Co-culturing hFB or HSC with CCA cell lines significantly increases navitoclax-induced apoptosis. *A*, expression of PDGF-B and PDGF-D was quantified in the human CCA cell lines KMCH, KMBC, HuCCT-1, and Mz-ChA-1 and the human cholangiocyte cell line H69 by quantitative real-time-PCR. Mean ± S.E. are depicted for *n* = 3. *, *p* < 0.05; **, *p* < 0.01; ***, *p* < 0.001 *versus* H69. *B*, two hFB cell lines and HSC were co-cultured with each of the four CCA cell lines. HSC were also co-cultured with H69 cells. After 24 h, navitoclax (*Nav*) or vehicle (*Veh*) was added for an additional 48 h. Apoptosis was assessed by DAPI staining and fluorescence microscopy. Means ± S.E. are depicted for *n* = 3. *, *p* < 0.05; **, *p* < 0.01; ***, *p* < 0.001. *C*, conditioned media from the human CCA cell lines KMCH, KMBC, HuCCT-1, and Mz-ChA-1 was added to quiescent hFB and HSC for 24 h. Navitoclax or vehicle was subsequently added for 48 h. Apoptosis was assessed by fluorometric measurement of caspase-3/7 activity. Means ± S.E. are depicted for *n* = 3. *, *p* < 0.05; **, *p* < 0.01; ***, *p* < 0.001. *D*, HSC cells with shRNA-targeted knockdown of the PDGF receptor, PDGFRB (Western blotting for PDGFRB in empty vector (*EV*) and shPDGFRB HSC cells; *left panel*) were co-cultured with four human CCA cell lines (KMCH, KMBC, HuCCT-1, Mz-ChA-1). After 24 h, navitoclax or vehicle was added for an additional 48 h. Apoptosis was assessed by DAPI staining and fluorescence microscopy (*right panel*). Means ± S.E. are depicted for *n* = 3. *E*, HSC cells with shRNA-targeted knockdown of the PDGF receptor, PDGFRB, were treated with conditioned media from four human CCA cell lines (KMCH, KMBC, HuCCT-1, Mz-ChA-1). After 24 h, navitoclax or vehicle was added for an additional 48 h. Apoptosis was assessed by fluorometric measurement of caspase-3/7 activity. Means ± S.E. are depicted for *n* = 3. * *p* < 0.05. *F*, HSC cells were co-cultured with four human CCA cell lines (KMCH, KMBC, HuCCT-1, Mz-ChA-1). Antibody neutralizing PDGFRB was added to the respective groups. After 24 h, navitoclax or vehicle was added for an additional 48 h. Apoptosis was assessed by DAPI staining and fluorescence microscopy (*right panel*). Mean ± S.E. are depicted for *n* = 3. *, *p* < 0.05; ***, *p* < 0.001.

Having observed that PDGF induces apoptotic priming, we sought to determine whether this sensitization could be replicated by co-culture of hFB and HSC with CCA cell lines. We first assessed expression of PDGF ligands in four different human CCA cell lines. Both PDGF-B and PDGF-D were abundantly expressed in all four CCA cell lines (Fig. 3*A*). Next, quiescent hFB and HSC were co-cultured with these four CCA cell lines and subsequently treated with navitoclax. Apoptotic sensitization, similar to that observed with PDGF, was observed in myofibroblast cells co-cultured with the CCA cell lines but not with H69 cells, which do not express PDGF-B or -D (Fig. 3*B*). Similarly, conditioned medium from CCA cell lines sensitized myofibroblasts to navitoclax-induced cell death (Fig. 3*C*).

Both PDGF-B and PDGF-D have high affinity for the receptor PDGFRB (39). The observed sensitization to apoptosis by co-culture with CCA cells or treatment with conditioned media from CCA cells was abrogated both in myofibroblasts with short hairpin RNA (shRNA)-targeted knockdown of PDGFRB or with the addition of antibody neutralizing PDGFRB (Fig. 3, *D–F*). Taken together, these data strongly suggest that PDGF can mediate apoptotic priming in myofibroblasts.

### PDGF Increases Cellular Levels of Puma in Myofibroblasts

**FIGURE 4.**
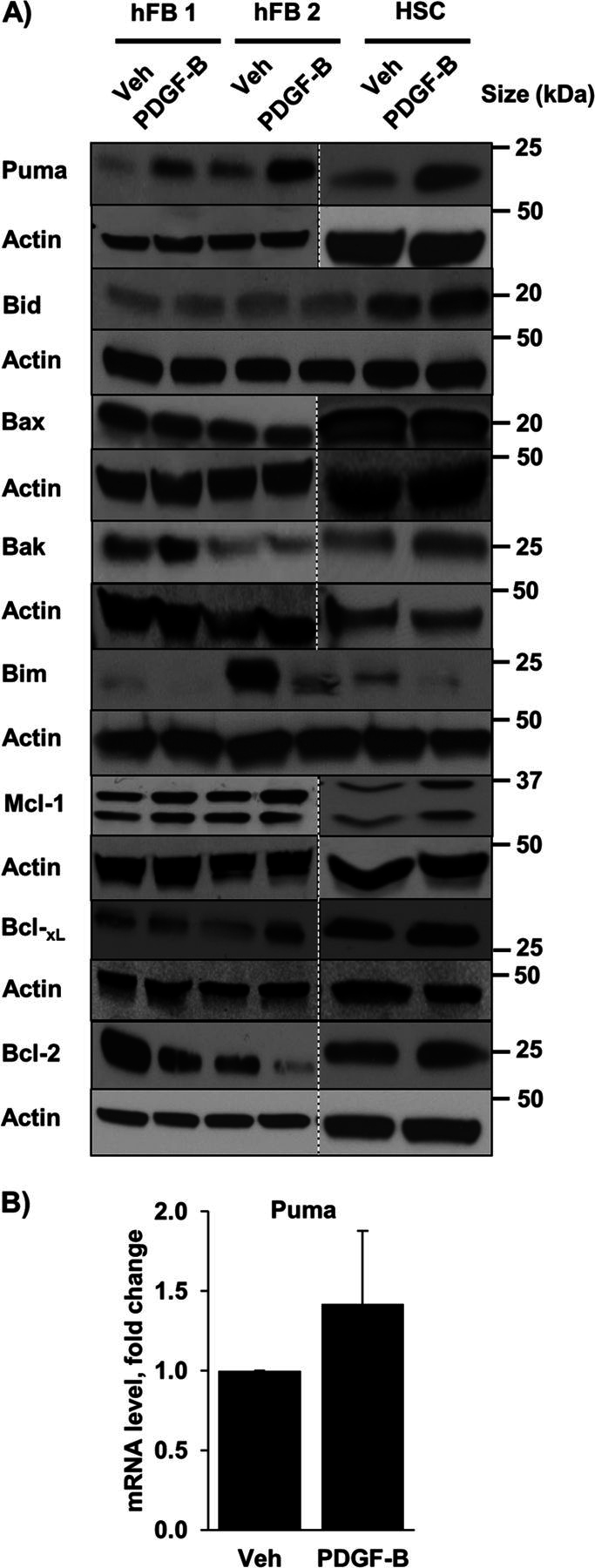
PDGF increases cellular Puma levels. *A*, whole cell lysates were prepared from quiescent hFB and HSC treated with vehicle (*Veh*) or PDGF-B for 24 h. Cell lysates were subject to immunoblot analysis of the Bcl-2 family of proteins. Actin was used as a loading control. Except where indicated by *dashed lines*, all lanes were adjacent on the membranes. *B*, expression of Puma was assessed by quantitative real-time-PCR in HSC. Means ± S.E. are depicted for *n* = 3.

To determine whether modulation of Bcl-2 proteins is responsible for the observed myofibroblast priming for cell death, we profiled the pro-apoptotic Bcl-2 proteins Bax and Bak, the antiapoptotic Bcl-2 proteins Mcl-1, Bcl-2, and Bcl-x_L_, and the BH3-only proteins Bim, Bid, and Puma by immunoblot analysis. Increased expression of the pro-apoptotic BH3-only protein Puma was consistently observed in PDGF-B-treated cells compared with vehicle-treated cells (Fig. 4*A*). Puma mRNA levels were similar in PDGF-B and vehicle-treated cells (Fig. 4*B*), suggesting that Puma up-regulation might reflect posttranslational differences. A PDGF-B-mediated decrease in Bcl-2 was also observed, albeit not consistently in all the myofibroblast cell lines.

### Bak Activation Leads to Navitoclax-triggered Apoptosis in CAF

**FIGURE 5.**
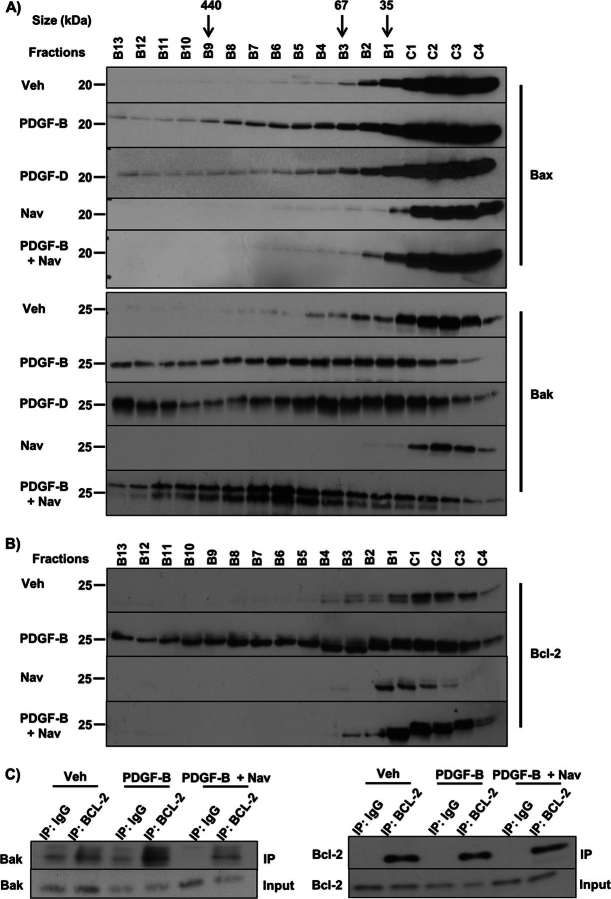
Navitoclax interferes with PDGF-facilitated binding of Bak and Bcl-2 triggering Bak oligomerization. *A*, HSC cells were treated with vehicle (*Veh*), PDGF-B, PDGF-D, navitoclax (*Nav*), and PDGF-B for 24 h followed by the addition of navitoclax for 24 h or PDGF-D for 24 h followed by the addition of navitoclax for 24 h. Subsequently, whole cell lysates were subjected to FPLC and fractions underwent immunoblot analysis for Bax and Bak. *B*, HSC cells were treated with vehicle, PDGF-B, navitoclax, or PDGF-B for 24 h followed by the addition of navitoclax for 24 h. Subsequently, whole cell lysates were subjected to FPLC, and fractions underwent immunoblot analysis for Bcl-2. *C*, immunoprecipitation (*IP*) of Bcl-2 from cell lysates of HSC cells treated as described in Fig. 5*B* and subsequent immunoblot analysis for Bak.

The mitochondrial pathway of cell death is triggered when either Bak or Bax oligomerizes in the mitochondrial outer membrane, causing mitochondrial outer membrane permeabilization with egress of apoptogenic mediators from the mitochondrial intermembrane space into the cytosol (16). Bak or Bax protein complexes in PDGF-B-treated cells were examined by fast protein liquid chromatography. Interestingly, we found that both PDGF-B alone and the combination of PDGF-B and navitoclax led to shifting of Bak from monomers to higher order protein complexes (Fig. 5*A*). As PDGF-B sensitizes to but does not trigger apoptosis, we hypothesized that PDGF-B induces binding of Bak to an antiapoptotic Bcl-2 family protein (Mcl-1, Bcl-2, Bcl-x_L_), accounting for the shift to a higher molecular weight. Indeed, a shift in Bcl-2 from monomers to larger-sized protein complexes occurred with PDGF-B treatment (Fig. 5*B*). In contrast, Mcl-1 and Bcl-x_L_ did not have a shift to larger-sized protein complexes with PDGF-B treatment (data not shown). Consistent with these results, PDGF-B treatment also resulted in increased association of Bak and Bcl-2, as indicated by co-immunoprecipitation of the endogenous proteins (Fig. 5*C*), and the addition of navitoclax disrupted this complex. These observations are consistent with a model in which PDGF-B facilitates binding of Bak to Bcl-2, leading to priming for cell death, and navitoclax interferes with this binding to trigger PDGF-primed apoptosis.

### PDGF-mediated Apoptotic Priming Is Bak- and Puma-dependent

**FIGURE 6.**
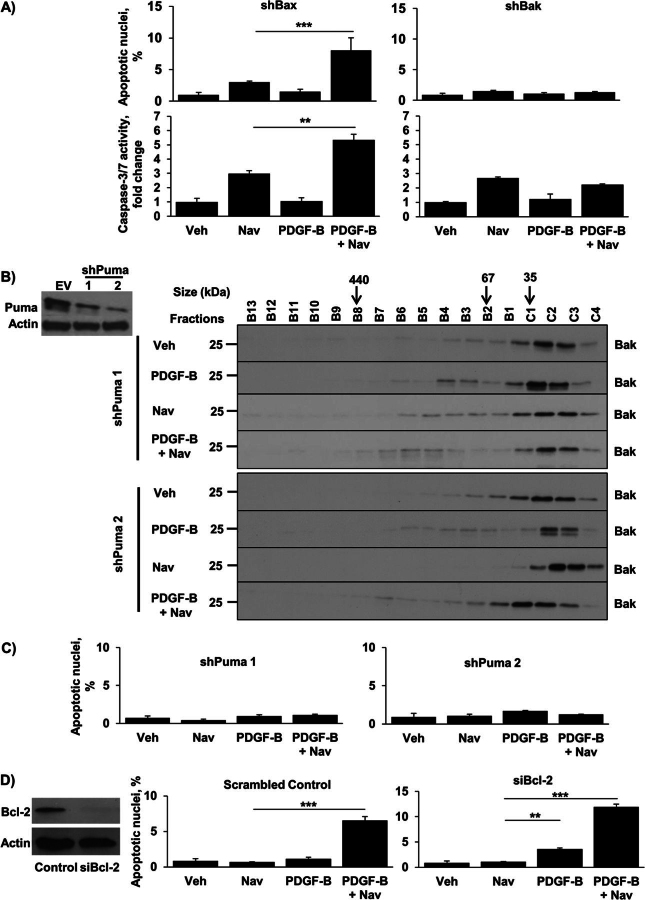
Knockdown of Puma or Bak, but not Bax, confers resistance to PDGF-mediated sensitization to navitoclax-induced apoptosis. *A*, Bak and Bax were knocked down in HSC cells by shRNA technique, and cells were treated as described in Fig. 2*A*. Apoptosis was quantified using DAPI staining plus fluorescence microscopy (*upper panels*) and by measuring caspase-3/7 activity (*lower panels*). Means ± S.E. are depicted for *n* = 3. **, *p* < 0.01; ***, *p* < 0.001. *Veh*, vehicle; *Nav*, navitoclax. *B*, Puma was knocked down in HSC cells by shRNA technique (Western blotting for Puma in empty vector (*EV*) and shPuma HSC cells; *left panel*). shPuma HSC cells (clone 1 and 2) were treated as described in Fig. 5*B* and subjected to FPLC, and fractions subsequently underwent immunoblot analysis for Bak (*right panel*). *C*, shPuma cells (*clones 1* and *2*) were treated as described in Fig. 2*A*. Apoptosis was quantified using DAPI staining plus fluorescence microscopy. Means ± S.E. are depicted for *n* = 3. *D*, cells transfected with a specific siRNA for Bcl-2 (Western blotting for Bcl-2; *left panel*) were treated as described in Fig. 5*B*. Cells transfected with scramble siRNA were used as a control. Apoptosis was quantified using DAPI staining plus fluorescence microscopy. Means ± S.E. are depicted for *n* = 3.

Although Bax and Bak are often viewed as being functionally equivalent, the changes observed in Fig. 5*A* suggested that Bak might play a more dominant role in PDGF-induced priming of CAFs. To test this possibility, myofibroblasts with shRNA-mediated knockdown of Bak or Bax, generated as previously described (3), were treated with PDGF-B with and without navitoclax. As indicated in Fig. 6*A*, Bax knockdown did not affect PDGF-B sensitization of myofibroblasts to cell death. In contrast, Bak knockdown markedly diminished the ability of navitoclax to induce apoptosis in PDGF-B treated shBak myofibroblasts (Fig. 6*A*), suggesting a critical role for Bak.

Because Puma can directly activate Bak (40, 41), we postulated that the PDGF-induced Puma up-regulation might be responsible for Bak activation and binding to Bcl-2. To test this prediction, myofibroblasts with shRNA-mediated knockdown of Puma were treated with PDGF-B and navitoclax (Fig. 6, *B*, and *C*). Knockdown of Puma prevented both the formation of higher order Bak protein complexes (Fig. 6*B*) and the induction of apoptosis by the PDGF-B/navitoclax combination (Fig. 6*C*), placing Puma upstream of Bak activation. Because PDGF mediates a decrease in Bcl-2 in some albeit not all myofibroblast cell lines, we also employed siRNA-mediated knockdown of Bcl-2 to determine its role in PDGF-induced apoptotic sensitization. Interestingly, myofibroblasts with knockdown of Bcl-2 displayed a modest increase in apoptosis with PDGF-B alone, and as expected a significant increase with the PDGF-B plus navitoclax combination was observed (Fig. 6*D*). Collectively, these observations suggest PDGF-B primes CAF for cell death primarily via a Puma-dependent mechanism, although down-regulation of Bcl-2 may also contribute to this altered phenotype.

### ABT-199 Functions Similar to Navitoclax in Sensitized Myofibroblasts

**FIGURE 7.**
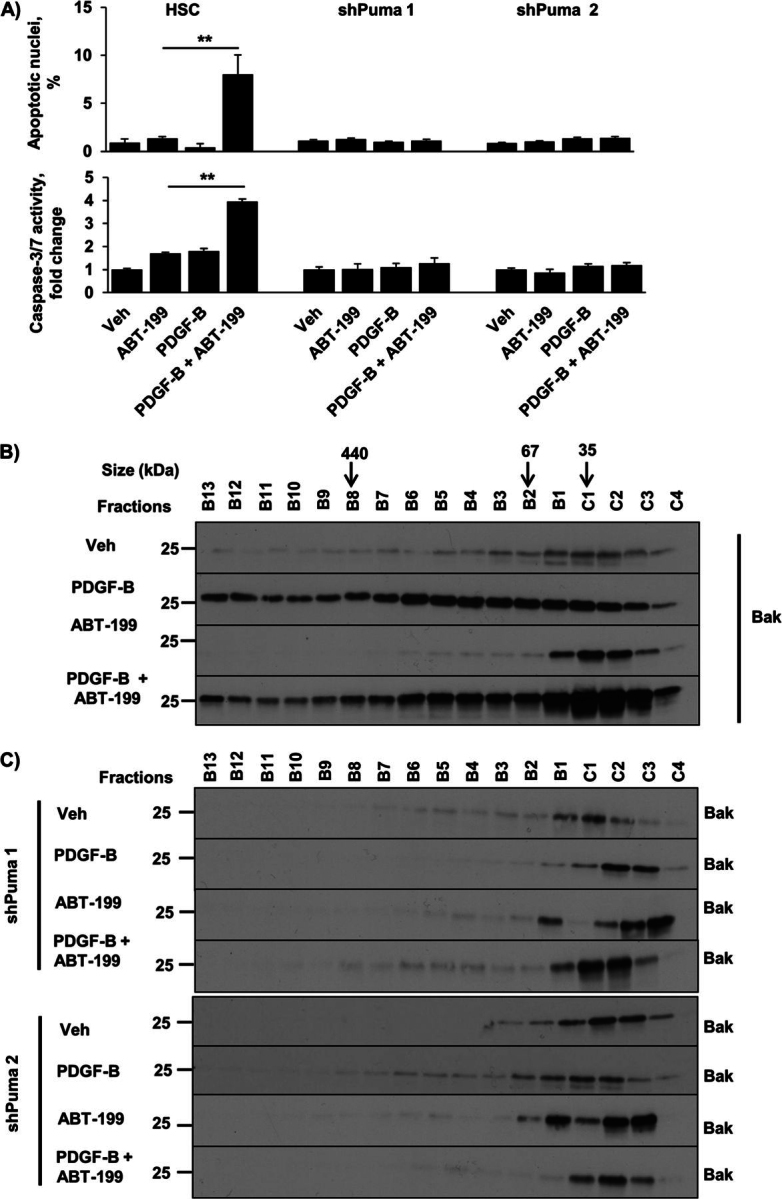
ABT-199 induces apoptosis in sensitized myofibroblasts in a Bak- and Puma-dependent manner. *A*, HSC and shPuma (*clones 1* and *2*) cells were treated as described in Fig. 2*A*. Apoptosis was quantified using DAPI staining plus fluorescence microscopy (*top panel*) and by measuring caspase-3/7 activity (*bottom panel*). *Veh*, vehicle. Means ± S.E. are depicted for *n* = 3. **, *p* < 0.01. *B*, HSC cells were treated with vehicle, PDGF-B for 24 h, ABT-199 for 24 h, and PDGF-B for 24 h followed by the addition of ABT-199 for 24 h. Subsequently, whole cell lysates were subjected to FPLC, and fractions underwent immunoblot analysis for Bak. *C*, shPuma HSC cells were treated as described in *B*. Subsequently, whole cell lysates were subjected to FPLC, and fractions underwent immunoblot analysis for Bak.

ABT-199, a BH3 agonist being developed in clinical trials, is structurally similar to navitoclax but differs from it functionally in that it only binds Bcl-2 and not Bcl-x_L_ (42)_._ We observed that ABT-199 also triggers apoptosis in myofibroblasts after PDGF-B treatment (Fig. 7*A*), similar to navitoclax (Fig. 2*A*). This observation suggests a functional role for the Bcl-2/Bak oligomers identified in Fig. 5 by suggesting a cytoprotective function for Bcl-2 in the PDGF-induced primed state. Similar to navitoclax, the combination of PDGF-B and ABT-199 also led to shifting of Bak from monomers to higher order protein complexes (Fig. 7*B*). Finally, we demonstrated that knockdown of Puma prevented both the formation of higher order Bak protein complexes (Fig. 7*C*) and the induction of apoptosis by the PDGF-B/ABT-199 combination (Fig. 7*A*). These data indicate that ABT-199 has a pharmacologic effect virtually identical to navitoclax in PDGF-primed myofibroblasts.

### ABT-199 Reduces Tumor Burden in a Murine Model of Cholangiocarcinoma

**FIGURE 8.**
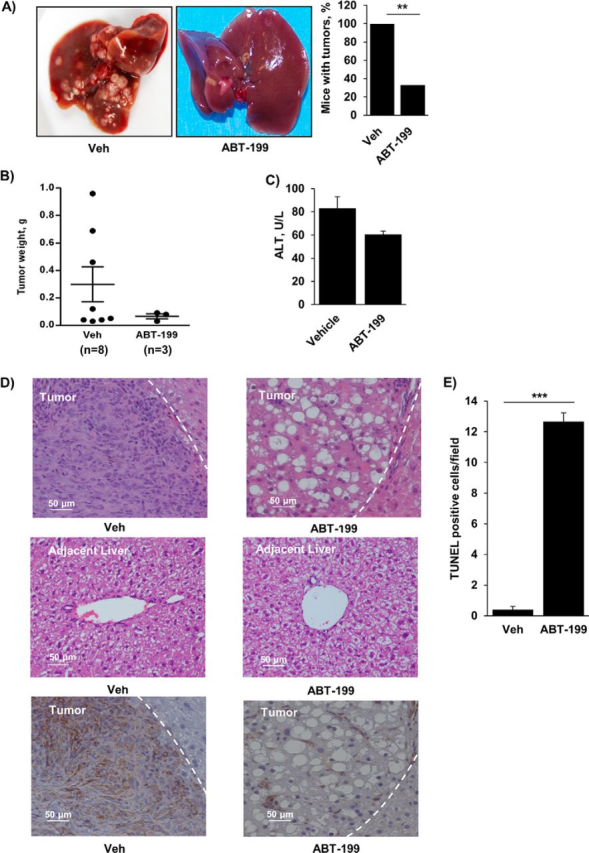
ABT-199 reduces tumor burden in a murine model of cholangiocarcinoma. *A*, mice having undergone biliary oncogene transduction, systemic IL-33 administration, and subsequent treatment with vehicle (*Veh*) or ABT-199 (50 mg/kg/day) were euthanized and examined for the presence of tumors and tumor burden. The mean is depicted for *n* = 8 (vehicle) and *n* = 9 (ABT-199). **, *p* < 0.01. *B*, tumors, if present, were carefully excised from the liver and weighed. *C*, serum alanine aminotransferase values were measured by standard techniques in samples from mice treated with vehicle and ABT-199. *D*, representative photomicrograph of hematoxylin and eosin-stained tumor sections and adjacent liver are shown (*upper* and *middle panels*, respectively). Characteristic tumor areas are to the *left of the dashed line*. Immunohistochemistry was used to detect α-SMA expression in tumors (*lower panels*). Original magnification. 20×. *E*, apoptotic cells were quantified by counting TUNEL-positive nuclei in 5 random microscopic fields (63×) using a confocal microscope. Means ± S.E. are depicted. ***, *p* < 0.001.

We previously demonstrated that navitoclax reduces tumor burden in a syngeneic orthotopic rodent model of CCA (3). We now sought to determine whether ABT-199, a newer BH3 mimetic with an improved safety profile, had a similar therapeutic effect in a more robust model of CCA. We recently generated a murine model of CCA combining ectopic oncogene expression with constitutively active AKT and YAP (Yes-associated protein) in the biliary tree combined with systemic IL-33 administration (35). In this model α-SMA-expressing myofibroblasts first appear ∼6 weeks after biliary oncogene transduction (data not shown). Hence, to examine a potential therapeutic effect of ABT-199, mice were treated with this agent for 2 weeks beginning on week 4 after oncogenic transformation. ABT-199 significantly reduced tumor formation compared with vehicle (Fig. 8*A*). Moreover, in the mice that did have tumor formation, ABT-199 reduced the tumor burden (Fig. 8*B*). Serum alanine aminotransferase values were similar in both groups, suggesting that ABT-199 did not induce significant injury in the adjacent liver (Fig. 8*C*). Histological examination of the adjacent liver also confirmed the absence of tissue injury in the ABT-199-treated group (Fig. 8*D*). Histologically, tumors occurring in mice that had received ABT-199 were less desmoplastic with a reduction in myofibroblasts and had decreased α-SMA expression compared with tumors in the vehicle group (Fig. 8*D*). Furthermore, these tumors had hollow, acellular areas that likely represented areas of myofibroblast depletion. Significant apoptosis within the tumors, as evidenced by an increase in TUNEL-positive cells, occurred with ABT-199 (Fig. 8*E*). These apoptotic cells likely represented both the initial myofibroblast death as well as secondary cancer cell death. These data are consistent with a chemotherapeutic role for ABT-199 via depletion of myofibroblasts from the tumor microenvironment of a highly desmoplastic malignancy.

## DISCUSSION

This study provides new mechanistic insights regarding CAF apoptotic priming by PDGF. These data indicate that 1) PDGF in the tumor microenvironment sensitizes CAF to apoptosis through changes in cellular Puma levels, 2) PDGF-mediated increases in Puma result in downstream formation of Bcl-2/Bak protein complexes, and 3) these complexes can be disrupted by BH3 mimetics, inducing Bak-dependent cell death. These findings are discussed in greater detail below.

Similar to activated T-cells in immune response and activated myofibroblasts in liver fibrosis, CAFs represent an activated state in desmoplastic cancers such as cholangiocarcinoma (11., 12., 13.). As such, CAF have an enhanced susceptibility to apoptosis (3). The biochemical basis for this effect has remained obscure. Here we have observed that PDGF isomers abundantly expressed by CCA cells sensitize myofibroblasts, but not CCA cells, to apoptosis. Priming for BH3 mimetic-induced cell death was not observed with other growth factors expressed in CCA, thereby demonstrating specificity of this effect of PDGF. These observations are also concordant with prior publications suggesting that PDGF may directly induce apoptosis in murine fibroblasts (43). Thus, despite being a growth factor, PDGF may also sensitize cells to apoptosis, an observation analogous to the concept of oncogene-induced cell death (44).

A unified model of Bcl-2 protein interactions in the mitochondrial pathway of apoptosis was recently proposed (17). In this unified model, two “modes” enable the antiapoptotic repertoire to block mitochondrial outer membrane permeabilization, a critical step in the mitochondrial pathway of cell death. The antiapoptotic Bcl-2 proteins sequester the direct-activator BH3-only proteins in MODE 1 and Bax and Bak in MODE 2. Under conditions of high stress or upon apoptotic stimulation, direct activators such as Bid, Bim, and Puma directly bind Bak or Bax, inducing changes conducive to their ability to permeabilize the outer mitochondrial membrane (17, 32, 45, 46). Antiapoptotic proteins such as Mcl-1, Bcl-2, and Bcl-x_L_ may in turn sequester the activated effectors, precluding their oligomerization. The present work indicates that PDGF is an apoptotic stimulus leading to increases in levels of the direct activator Puma in CAF. This in turn prompts changes in Bak, leading to its subsequent binding to the antiapoptotic protein Bcl-2. Although these PDGF-induced changes in Puma and Bak are not sufficient to induce apoptosis by themselves, the BH3 mimetic navitoclax functions as a derepressor and releases Bak from Bcl-2, allowing apoptosis. This model, which suggests that Bcl-2 is acting in MODE 2 to simultaneously prevent PDGF-induced apoptosis but leave the cell in a primed state, is consistent with the changes in Bak oligomerization observed under various conditions (FIGURE 5., FIGURE 6.) as well as the effects of PDGFRB, Bak, and Puma knockdowns (FIGURE 3., FIGURE 6.).

Bak and Bax often are redundant in the mitochondrial pathway of cell death, especially in fibroblasts (47). Hence, our observation that Puma priming of CAF for cell death was Bak-dependent was unexpected, especially because Puma can activate either Bak or Bax (40, 41). Additionally, in our prior studies using CAF, Bax was found to participate in the priming phenomenon (3). Close examination of Fig. 5*A* indicates that PDGF causes a small amount of Bax to form oligomers as well. However, the extent of Bax oligomerization is lower than the extent of Bak oligomerization. These observations suggest PDGF induces a specific cellular context in regard to apoptotic priming and that other priming phenomena yet to be defined may engage Bax. The relative roles of these two apoptotic mediators in CAF apoptotic priming *in vivo* will require further mechanistic studies.

The current data suggest that PDGF-B signaling pathways increase cellular levels of Puma, which in turn, is essential for Bak activation and apoptosis priming. The increase in Puma appears to be independent of transcription, as steady state mRNA Puma expression in myofibroblasts was unaltered by PDGF-B treatment. Although decreases in microRNA 221, 222, 296, and 483 could potentially explain enhanced protein transcription in the absence of transcriptional activation (49., 50., 51.), we did not observe changes in these microRNA after exposure to PDGF-B (data not shown). Perhaps loss of other yet to be examined microRNA are responsible for the increase in Puma. We are currently exploring this concept. Phosphorylation of Puma by MAPK and IκB kinase (IKK) may decrease protein stability (48), suggesting that phosphatases may enhance its stability. The precise phosphatase responsible for this effect is unclear and remains to be determined in future investigations.

Our data suggesting a protective role for Bcl-2 in PDGF-mediated CAF apoptotic priming prompted investigation of a newer BH3 mimetic, ABT-199. Navitoclax, the first orally bioavailable Bcl-2 family inhibitor, inhibits Bcl-2 and Bcl-x_L._ Inhibition of the latter results in dose-limiting thrombocytopenia (18). ABT-199, a potent Bcl-2 inhibitor, was subsequently designed in an effort to spare platelets and allow higher circulating levels of the drug in patients with hematopoietic malignancies. It showed significant promise in preclinical studies by induction of apoptosis in hematological cancer cell lines and tumor regression *in vivo* (42) and is currently being evaluated in clinical trials. We have now demonstrated a proapoptotic effect of ABT-199 on myofibroblasts primed for apoptosis. ABT-199 was found to be mechanistically similar to navitoclax in our model; hence, its proapoptotic effect was also Bak- and Puma-dependent. Additionally, ABT-199 reduced tumor formation and tumor burden in a murine model of CCA via depletion of myofibroblasts from the tumor stroma. Accordingly, targeting the tumor stroma in desmoplastic malignancies represents a novel and promising role for this new BH3 mimetic. Further preclinical studies are warranted to determine the efficacy of ABT-199 in desmoplastic malignancies.

In summary, the current study advances our understanding of both CAF biology and cellular priming for apoptosis. The present observations implicate PDGF-B and -D isoforms in inducing CAF priming for cell death in the tumor microenvironment. These PDGF isoforms induce this altered cellular state by increasing levels of the proapoptotic protein Puma. These observations raise the possibility that PDGF and/or Puma expression in desmoplastic tumors might serve as biomarkers for this priming phenomenon. In addition, our data provide new insight into the primed state. Previously, BH3 mimetics such as navitoclax were thought to displace activator BH3 proteins from antiapoptotic Bcl-2 proteins, thereby liberating them to activate Bak or Bax. The current observations based on FPLC and immunoprecipitation of protein complexes suggest that BH3-mimetics might kill primed cells by disrupting preformed complexes between antiapoptotic Bcl-2 proteins and activated Bak or Bax, permitting the latter to execute the cell death program. Furthermore, these data suggest that BH3 mimetics may be therapeutic in desmoplastic cancers by deleting CAF, a concept warranting further explanation in preclinical and perhaps clinical studies.
